# Professional identity, academic satisfaction, and career commitment among undergraduate nursing students in India: a cross-sectional study

**DOI:** 10.3389/fpsyg.2026.1931177

**Published:** 2026-08-19

**Authors:** Ramya Kundayi Ravi, Shivani Sharma, Renitha MSJ, Mohamed Anas Patni, Bhoomendra A. Bhongade, Priya Baby

**Affiliations:** 1 RAK College of Nursing, RAK Medical and Health Sciences University, Ras Al Khaimah, United Arab Emirates; 2 SPHE College of Nursing, Saraswati Group of Colleges, Mohali, Punjab, India; 3 St. Joseph’s College of Nursing, Kothamangalam, Kerala, India; 4 Department of Community Medicine, RAK College of Medical Sciences, Ras Al Khaimah Medical and Health Science University, Ras Al Khaimah, United Arab Emirates; 5 Department of Pharmaceutical Chemistry, RAK College of Pharmacy, RAK Medical and Health Sciences University, Ras Al Khaimah, United Arab Emirates; 6 College of Nursing, NIMHANS, Bangalore, India

**Keywords:** academic satisfaction, career commitment, cluster analysis, India, leadership involvement, mediation analysis, nursing students, professional identity

## Abstract

**Introduction:**

Professional identity, academic satisfaction, and career commitment are important psychological and educational factors that may influence nursing students’ long-term engagement with the nursing profession. In countries such as India, where nursing workforce retention and migration are major concerns, understanding these factors is essential for strengthening future workforce stability. This study examined the levels of professional identity, academic satisfaction, and career commitment among undergraduate nursing students in India, identified student profiles based on these constructs, and examined whether academic satisfaction showed a statistical indirect association in the relationship between professional identity and career commitment.

**Methods:**

A cross-sectional correlational study was conducted among 354 undergraduate nursing students from two nursing colleges in India. Participants were recruited using convenience sampling. Data were collected using structured, validated self-administered questionnaires measuring professional identity, academic satisfaction, and career commitment. Descriptive statistics, Pearson correlation, k-means cluster analysis, mediation analysis using PROCESS Model 4, and multiple linear regression were performed.

**Results:**

Students reported high professional identity (mean = 66.98, SD = 8.78) and academic satisfaction (mean = 19.15, SD = 3.67), but moderate career commitment (mean = 12.79, SD = 2.16). Professional identity was positively correlated with academic satisfaction (r = 0.517, *p* < 0.001) and career commitment (*r* = 0.224, *p* < 0.001). Academic satisfaction was also positively correlated with career commitment (*r* = 0.283, *p* < 0.001). Cluster analysis identified three student profiles: low identity and satisfaction profile, high identity, satisfaction, and commitment profile, and higher identity/satisfaction but lower commitment profile. PROCESS analysis showed a significant indirect association between professional identity and career commitment through academic satisfaction, (B = 0.0378, 95% CI: 0.0165–0.0607). In regression analysis, professional identity, academic satisfaction, and leadership involvement were significantly associated with career commitment. The model explained 11.9% of the variance in career commitment.

**Conclusion:**

Career commitment among nursing students was associated with professional identity, academic satisfaction, and leadership involvement. However, the modest explained variance suggests that additional personal, educational, and contextual factors should be explored. Longitudinal and multicenter studies are recommended to better understand the development of career commitment among nursing students.

## Introduction

Developing and retaining a committed nursing workforce is a global priority amid persistent nursing shortages, increasing healthcare demands and workforce mobility. The World Health Organization (WHO) has estimated a global shortage of approximately 5.9 million nurses, with the greatest deficits concentrated in low- and middle-income countries.(Kiptulon et al., 2025) Although nursing education has expanded substantially in many countries, retaining graduates within the profession remains a major challenge due to career transitions, turnover intentions and international migration.(Laari et al., 2024) India is among the largest suppliers of nurses to the global workforce, and migration to higher-income countries continues to raise concerns regarding workforce retention and sustainability.(Ravi et al., 2025; Walton-Roberts et al., 2025) Consequently, understanding the factors that influence nursing students’ commitment to the profession is critical, as career intentions formed during training often shape future workforce participation and retention.

Previous research has identified professional identity, academic experiences, and career commitment as key factors influencing nursing students’ professional development and career trajectories. A strong professional identity has been associated with greater engagement, motivation, resilience, and intention to remain in nursing (Koch, 2014; Chen et al., 2025). Similarly, positive educational experiences and academic satisfaction are linked to improved learning outcomes, persistence, and professional growth (Geok Lim, 2016). However, despite generally favorable levels of professional identity and academic satisfaction, studies have frequently reported only moderate levels of career commitment among nursing students, raising concerns about future retention in the profession (Ravi et al., 2025; Ding et al., 2026; Tsai et al., 2026).

This concern is particularly relevant in India, where increasing healthcare demands coexist with challenges related to workforce shortages, uneven distribution of nurses, and growing international migration opportunities for nurses (Laari et al., 2024; Walton-Roberts et al., 2025). While previous studies have examined professional identity, academic satisfaction, and career commitment independently, limited evidence exists regarding the mechanisms through which these factors influence each other (Johnson, 2018; Mukherjee et al., 2024; Pandya et al., 2024). In particular, little is known about whether academic satisfaction serves as a pathway linking professional identity to career commitment among nursing students in India. Furthermore, most existing studies have adopted variable-centered approaches, potentially overlooking distinct student subgroups that may differ in their professional development and career intentions (Mukherjee et al., 2024).

Understanding these relationships is essential for developing educational strategies that not only enhance students’ academic experiences but also strengthen their long-term commitment to nursing careers. Therefore, this study aimed to examine the levels of professional identity, academic satisfaction, and career commitment among undergraduate nursing students in India, identify distinct student profiles based on these constructs, examine the predictors of career commitment, and investigate the mediating role of academic satisfaction in the relationship between professional identity and career commitment.

### Conceptual framework

This study was guided by a mediation framework proposing that professional identity influences career commitment directly and indirectly through academic satisfaction among undergraduate nursing students. The framework draws from the theoretical perspectives of Professional Socialization theory. According to this theory, a well internalized professional identity shapes the student’s appraisal of their educational environment. This influences the meaningful interpretation of their educational experiences influences their academic satisfaction (Zarshenas et al., 2014; Sadeghi Avval Shahr et al., 2019). In this study, professional identity is conceptualized as the internalization of professional values, roles, and self-perceptions as a future nurse, measured using the Professional Identity Scale for Nursing Students, which encompasses professional self-image, benefit of retention and risk of turnover, social comparison and self-reflection, independence of career choice, and social modeling (Hao et al., 2014). Professional identity is considered a key outcome and has been reported to be associated with professional commitment, career intentions, and retention (Baldwin et al., 2014; Chen et al., 2025).

Academic satisfaction, the mediator, represents students’ overall evaluation of their educational experiences, including satisfaction with their nursing programs, learning environments, and professional preparations. Previous studies have shown that positive academic experiences enhance engagement, motivation, and persistence in nursing students (Geok Lim, 2016; Laari et al., 2024; Kundayi Ravi et al., 2026). Students with a stronger professional identity tend to report greater satisfaction with their educational journey, suggesting that academic satisfaction may serve as a mechanism linking professional identity to professional outcomes (Jung, 2020).

The self -determination theory explains the translation of academic satisfaction to career commitment (Ryan and Deci, 2017). It proposes that motivation and commitment are strongest when the environment supports the needs of competence, relatedness and satisfaction. On this basis academic satisfaction is expected to strengthen career commitment (Wang et al., 2019) Career commitment is conceptualized as an individual’s psychological attachment to and intention to remain in the nursing profession. Measured using Blau’s Career Commitment Scale, it reflects the desire to continue pursuing nursing as a long-term career (Blau, 1985). Higher career commitment has been associated with a stronger professional identity, positive educational experiences, professional values, and a sense of calling (Chen et al., 2025; Ding et al., 2026; Tsai et al., 2026).

Based on existing evidence, the framework proposes that students with a stronger professional identity are more likely to experience greater academic satisfaction, which subsequently enhances career commitment. This proposition is supported by studies indicating that educational experiences play a critical role in translating professional values and identity into a sustained commitment to nursing careers (Chen et al., 2025; Ding et al., 2026). While prior research has examine professional identity, career commitment and academic satisfaction as independent correlates, this study integrates professional socialization theory and self-determination theory to test a theory-derived indirect pathway. This extends beyond correlational evidence to an explanatory mechanism. The framework is particularly relevant in the Indian context, where workforce shortages, migration aspirations, and evolving professional expectations may influence nursing students’ long-term commitment to the profession. The following hypotheses were developed and tested as part of this study.

*H1*: Professional identity is positively associated with career commitment among undergraduate nursing students.

*H2*: Professional identity is positively associated with academic satisfaction among undergraduate nursing students.

*H3*: Academic satisfaction is positively associated with career commitment among undergraduate nursing students.

*H4*: Academic satisfaction shows a significant statistical indirect association in the relationship between professional identity and career commitment among undergraduate nursing students.

## Materials and methods

### Study design and setting

This study employed a cross-sectional correlational design. This study was reported in accordance with the Strengthening the Reporting of Observational Studies in Epidemiology (STROBE) guidelines (Supplementary file 1) (Cuschieri, 2019). The study was conducted in two selected nursing colleges in India, conveniently selected from the northern and southern regions. Both institutions offer undergraduate and graduate nursing programs under the Indian Nursing Council.

### Participants and sampling

The study population comprised undergraduate nursing students enrolled in the selected institutions during the 2025–2026 academic year. A convenience sampling approach was adopted to recruit the study participants, whereby all students enrolled in the Bachelor of Science in Nursing (BSc Nursing) program from the first to the fourth year of study were invited to participate. Undergraduate nursing students currently enrolled in the nursing program, and willing to provide informed consent were eligible for inclusion. Students who did not provide consent or submit incomplete questionnaires were excluded from the final analysis.

### Sample size estimation

The sample size was estimated using G*Power version 3.1 for multiple linear regression, as career commitment was the primary outcome variable and the study aimed to identify its predictors [18]. The following parameters were used: test family = F tests; statistical test = linear multiple regression: fixed model, *R*^2^ deviation from zero; type of power analysis = *a priori*; effect size f^2^ = 0.05; *α* error probability = 0.05; statistical power = 0.80; and number of predictors = 11. The assumed effect size of f^2^ = 0.05 was selected to detect a small-to-moderate association, which was considered appropriate for psychosocial and educational variables. The calculation yielded a minimum required sample size of 346 participants. The final sample included 354 undergraduate nursing students, which exceeded the minimum required sample size for the primary regression analysis. Mediation and exploratory K-means cluster analyses were performed as secondary analytical components, and the cluster findings were interpreted as exploratory because no separate a priori sample size calculation was performed for cluster analysis.

### Variables and measurements

Data were collected using a structured self-administered questionnaire comprising four sections (Supplementary file 2).

### Section one

Included structured items to collect the demographic and Academic Characteristics of students who participated in the study, including age, gender, year of study, area of residence, reasons for choosing nursing, intention to migrate after graduation, participation in voluntary social or community activities, and involvement in leadership roles within the institution.

### Section two

Professional identity was measured using the Professional Identity Scale for Nursing Students (PISNS) developed by Hao et al. (2014). The instrument consists of 17 items distributed across five domains: professional self-image, benefit of retention and risk of turnover, social comparison and self-reflection, independence of career choice, and social modeling. Responses were rated on a five-point Likert scale ranging from 1 (strongly disagree) to 5 (strongly agree). One item was reverse-scored. The total scores ranged from 17 to 85, with higher scores indicating a stronger professional identity. The original scale demonstrated good psychometric properties, with an overall Cronbach’s alpha of 0.83 and subscale reliability coefficients ranging from 0.62 to 0.82. Construct validity was established through confirmatory factor analysis. Internal consistency was excellent for the Professional Identity Scale (Cronbach’s alpha = 0.908) and the Academic Satisfaction Scale (Cronbach’s alpha = 0.924).

### Section 3

Academic satisfaction was assessed using five items adapted from the Satisfaction with Current Learning subscale of the National League for Nursing (NLN) Student Satisfaction and Self-Confidence in Learning Scale (Franklin et al., 2014). The items evaluated students’ satisfaction with their learning experiences, instructional methods, learning activities, teaching style, and educational materials. Responses were recorded on a five-point Likert scale ranging from 1 (strongly disagree) to 5 (strongly agree). The total scores ranged from 5 to 25, with higher scores indicating greater academic satisfaction. Previous studies have reported excellent internal consistency for the satisfaction subscale, with a Cronbach’s alpha of 0.94. The internal consistency of the Academic Satisfaction Scale was Cronbach’s alpha = 0.924.

### Section 4

Career commitment was measured using the four-item Career Commitment Scale developed by Blau. (Blau, 1985). The scale assesses an individual’s attachment to and commitment to their chosen profession. Responses were rated on a five-point Likert scale ranging from 1 (strongly disagree) to 5 (strongly agree). Two negatively worded items were reverse-coded before analysis. The total scores ranged from 4 to 20, with higher scores indicating a stronger commitment to the nursing profession. The original instrument demonstrated satisfactory reliability, with Cronbach’s alpha coefficients ranging from 0.79 to 0.85, and evidence of construct validity. The Career Commitment Scale showed acceptable internal consistency for a short four-item scale, with Cronbach’s alpha = 0.616.

### Data collection procedure

Data were collected between 1st March and 15th March 2026. An online survey was created using Google Forms and distributed to eligible students through institutional communication channels, including official student groups and class representatives at the university. The faculty members who were investigators in the study distributed the link in the classroom during their free period. No academic activities were disrupted during the data collection period. An information sheet describing the study objectives, procedures, voluntary nature of participation, and confidentiality measures was provided on the first page of the survey questionnaire. Electronic informed consent was obtained before the participants completed the questionnaire. Only consenting students were directed to complete the survey. To maintain anonymity, no personal identifiable information was collected from the participants. Students were informed of their right to decline participation or withdraw from the study at any time, without penalty. Anonymity and confidentiality were maintained throughout the study. All data were securely stored and used exclusively for research purposes.

### Ethical considerations

Ethical approval was obtained from the Institutional Ethics Committees of the SPHE College of Nursing, Mohali, Punjab, India (Approval No.: IEC/ SPHE/2026/209). The study followed all the guidelines and recommendations provided by the Declaration of Helsinki. Permission to conduct the study was obtained from the respective college administrations, and consent was obtained from all students who participated in the study.

### Statistical analysis

Data were analyzed using descriptive statistics to summarize the demographic characteristics and main study variables, including means, standard deviations, medians, and interquartile ranges. Pearson correlation coefficients were computed to examine the relationships among professional identity, academic satisfaction, and career commitment. Internal consistency of the study scales was assessed using Cronbach’s alpha. The reliability of short scales was interpreted cautiously, particularly when the scale included negatively worded items. An exploratory K-means cluster analysis was performed using standardized scores of professional identity, academic satisfaction, and career commitment to identify student subgroups. K-means clustering was selected because the aim was to develop an exploratory, interpretable person-centered classification using continuous scale scores. As the three scales had different score ranges, all variables were standardized as *Z*-scores before clustering. Two- and three-cluster solutions were examined and compared based on cluster size, convergence, interpretability of standardized cluster centers, parsimony, and educational meaningfulness. The three-cluster solution was retained because it provided a more differentiated and interpretable classification than the two-cluster solution. Raw mean scores were examined within clusters to aid profile interpretation. PROCESS Model 4 was used to estimate the statistical indirect association between professional identity and career commitment through academic satisfaction. Because of the cross-sectional design, this analysis was interpreted as statistical mediation only and not as evidence of a causal pathway. Multiple linear regression analysis was employed to identify significant predictors of career commitment, including professional identity, academic satisfaction, demographic variables and leadership involvement. The model fit was assessed using R^2^ and adjusted R^2^ values. Before inferential analyses, statistical assumptions were assessed. Normality of the summed scale scores was examined using histograms, Q–Q plots, and skewness/kurtosis values. Linearity between the main continuous variables was assessed using scatterplots and by comparing Pearson and Spearman correlations. For the multiple linear regression model, residual normality and multicollinearity were assessed before interpreting the final model. Pearson correlation was used because professional identity, academic satisfaction, and career commitment were calculated as summed multi-item scale scores and treated as approximately continuous variables. A *p*-value < 0.05 was considered statistically significant. All analyses were performed using the SPSS version 32.

## Results

A total of 354 undergraduate nursing students were included in the study. The mean age of the participants was 20.75 ± 2.27 years (Table 1). Most of them were female (86.4%) and intended to migrate after course completion (83.3%). More than half, 192 (54.2%) had not volunteered for non-mandatory social or community activities, and 239 (67.5%) were not involved in leadership roles.

**Table 1:** Profile of study participants.

Variable	Categories	*n* (%)
Age, years	Mean ± SD	20.75 +/− 2.27
	Median	21
	Range	17–39
Study region	North	184 (52.0)
	South	170 (48.0)
Gender	Female	306 (86.4)
	Male	48 (13.6)
Year of study	1st year	120 (33.9)
	2nd year	50 (14.1)
	3rd year	65 (18.4)
	4th year	119 (33.6)
Area of residence	Rural	236 (66.7)
	Urban	118 (33.3)
Reason for choosing nursing	Personal interest	189 (53.4)
	Job security	103 (29.1)
	Family influence	38 (10.7)
	Other	19 (5.4)
	Unable to get another course	5 (1.4)
Intention to migrate after course completion	Yes	295 (83.3)
	No	59 (16.7)
Volunteered for non-mandatory social/community activities	Yes	162 (45.8)
	No	192 (54.2)
Leadership role in college/university	Yes	115 (32.5)
	No	239 (67.5)
Perceived family economic status	Good/Satisfactory	274 (77.4)
	Not satisfactory	80 (22.6)

The mean professional identity score was 66.98 ± 8.78, signifying a high level of professional identity (Table 2). The mean academic satisfaction score was 19.15 ± 3.67, reflecting a relatively high satisfaction. The mean career commitment score was 12.79 ± 2.16, indicating a moderate level of commitment to the nursing profession. Internal consistency was excellent for professional identity and academic satisfaction, with Cronbach’s alpha values of 0.908 and 0.924, respectively. The Career Commitment Scale demonstrated modest internal consistency, Cronbach’s alpha = 0.616, which may be partly attributable to the short four-item structure and the inclusion of negatively worded items. Therefore, findings involving career commitment were retained but interpreted cautiously. Assumption checks showed acceptable distributional properties of the summed scale scores, and Pearson and Spearman correlations showed similar patterns of association; therefore, parametric analyses were considered appropriate.

**Table 2:** Descriptive statistics of main study variables.

Variable	No. of items	Possible score range	Observed score range	Mean ± SD	Median	IQR
Professional identity	17	17–85	42–83	66.98 ± 8.78	66.0	61.0–74.0
Academic satisfaction	5	5–25	5–25	19.15 ± 3.67	20.0	17.0–21.0
Career commitment	4	4–20	7–20	12.79 ± 2.16	12.0	12.0–14.0

Negatively worded items were reverse coded before calculating total scores. IQR, interquartile range.

Table 3 demonstrates that professional identity has a significant positive correlation with academic satisfaction (*r* = 0.517, *p* < 0.001) and career commitment (*r* = 0.224, *p* < 0.001). Academic satisfaction was also positively correlated with career commitment (*r* = 0.283, *p* < 0.001).

**Table 3:** Correlation between professional identity, academic satisfaction, and career commitment among undergraduate nursing students.

Variable	Professional identity	Academic satisfaction	Career commitment
Professional identity	1		
Academic satisfaction	0.517***	1	
Career commitment	0.224***	0.283***	1

Pearson correlation coefficients are shown. ***p < 0.001.

An exploratory K-means cluster analysis was conducted using the standardized total scores of professional identity, academic satisfaction, and career commitment (Table 4). In the two-cluster solution, students were broadly classified into a lower overall profile, 182 (51.4%) and a higher overall profile, 172 (48.6%). The lower overall profile showed lower standardized centers across professional identity, academic satisfaction, and career commitment, whereas the higher overall profile showed positive standardized centers across all three variables.

**Table 4:** Exploratory K-means cluster solutions based on professional identity, academic satisfaction, and career commitment.

Cluster solution	SPSS cluster	Profile label	Adjusted *n* (%)	Standardized cluster centers: PI/AS/CC	Raw mean scores: PI/AS/CC
2-cluster solution	1	Lower overall profile	182 (51.4)	−0.675/−0.627/−0.359	61.05/16.85/12.65
	2	Higher overall profile	172 (48.6)	0.706/0.665/0.377	73.18/21.58/14.72
3-cluster solution	1	Low identity and satisfaction profile	120 (33.9)	−0.994/−0.873/−0.226	58.26/15.95/13.03
	2	High identity, satisfaction, and commitment profile	78 (22.0)	0.705/0.913/1.374	73.17/22.49/17.51
	3	Higher identity/satisfaction but lower commitment profile	156 (44.1)	0.407/0.222/−0.509	70.55/19.96/12.23

The three-cluster solution provided a more meaningful classification. Cluster 1, comprising 120 students (33.9%), was characterized by low professional identity and academic satisfaction, with slightly below-average career commitment. Cluster 2 included 78 students (22.0%) and represented the most favorable profile, with high professional identity, high academic satisfaction, and the highest career commitment. Cluster 3 was the largest group, comprising 156 students (44.1%), and showed relatively higher professional identity and academic satisfaction but lower career commitment. The two-cluster solution produced broad lower and higher overall profiles, whereas the three-cluster solution provided a more meaningful classification: low identity and satisfaction profile, high identity/satisfaction/commitment profile, and higher identity/satisfaction but lower commitment profile. Therefore, the three-cluster solution was retained for interpretation.

PROCESS Model 4 was used to examine whether academic satisfaction showed a statistical indirect association in the relationship between professional identity and career commitment. Professional identity was significantly associated with academic satisfaction, B = 0.2159, *p* < 0.001. Academic satisfaction was significantly associated with career commitment after controlling for professional identity, B = 0.1749, p < 0.001. The direct association between professional identity and career commitment was not statistically significant after academic satisfaction was included in the model, B = 0.0335, *p* = 0.0796. The indirect association through academic satisfaction was statistically significant, B = 0.0378, 95% bootstrap CI: 0.0165–0.0607. Therefore, academic satisfaction may represent a statistical explanatory pathway linking professional identity and career commitment; however, causality cannot be inferred from these cross-sectional data (see Table 5).

**Table 5:** Mediation analysis of academic satisfaction in the relationship between professional identity and career commitment.

Path	Estimate	SE	*p*-value/95% CI
Path a: Professional identity → Academic satisfaction	0.2159	0.0191	*p* < 0.001
Path b: Academic satisfaction → Career commitment, controlling for professional identity	0.1749	0.0457	*p* < 0.001
Direct effect: Professional identity → Career commitment, controlling for academic satisfaction	0.0335	0.0191	*p* = 0.0796
Indirect effect: Professional identity → Academic satisfaction → Career commitment	0.0378	0.0111	95% CI: 0.0165 to 0.0607

The mediator model explained 26.8% of the variance in academic satisfaction (*R*^2^ = 0.268). The outcome model explained 8.8% of the variance in career commitment (*R*^2^ = 0.088).

The regression model included (Table 6) professional identity score, academic satisfaction score, age, gender, year of study, area of residence, reason for choosing nursing, intention to migrate after course completion, volunteering experience, leadership role, and perceived family economic status as predictors, with the career commitment score as the dependent variable. The overall regression model was statistically significant, *F*(11, 339) = 4.171, *p* < 0.001, indicating that the included predictors collectively explained a significant proportion of the variation in career commitment. The model explained 11.9% of the total variance, with an adjusted *R*^2^ of 0.091.

**Table 6:** Multiple linear regression analysis for predictors of career commitment among undergraduate nursing students.

Predictor	B	SE	*β*	95% CI for B	*t*	*p*-value
Professional identity	0.041	0.020	0.128	0.002 to 0.080	2.054	0.041^*^
Academic satisfaction	0.163	0.046	0.213	0.072 to 0.253	3.525	<0.001^*^
Age	−0.030	0.076	−0.024	−0.179 to 0.119	−0.392	0.695
Gender	−0.186	0.422	−0.023	−1.016 to 0.644	−0.441	0.659
Year of study	−0.247	0.136	−0.112	−0.515 to 0.021	−1.816	0.070
Area of residence	0.117	0.310	0.020	−0.493 to 0.727	0.376	0.707
Reason for choosing nursing	0.158	0.130	0.066	−0.097 to 0.413	1.219	0.224
Intention to migrate	−0.001	0.385	0.000	−0.759 to 0.757	−0.002	0.998
Volunteering experience	−0.315	0.304	−0.056	−0.913 to 0.282	−1.038	0.300
Leadership role	0.816	0.332	0.137	0.164 to 1.469	2.460	0.014^*^
Perceived family economic status	0.126	0.354	0.019	−0.570 to 0.822	0.356	0.722

Model summary: *R* = 0.345; *R*^2^ = 0.119; adjusted *R*^2^ = 0.091; *F*(11, 339) = 4.171; *p* < 0.001.

Professional identity was a significant positive predictor of career commitment (B = 0.041, 95% CI: 0.002–0.080, *p* = 0.041), indicating that higher professional identity scores were associated with higher career commitment. Academic satisfaction was also a significant positive predictor (B = 0.163, 95% CI: 0.072–0.253, *p* < 0.001). Students who reported involvement in leadership roles had significantly higher career commitment scores (B = 0.816, 95% CI: 0.164–1.469, *p* = 0.014).

Although the model was statistically significant, the adjusted *R*^2^ value indicated modest explanatory power; therefore, the identified predictors should be interpreted as partial contributors to career commitment rather than comprehensive determinants.

## Discussion

This study examined professional identity, academic satisfaction, and career commitment among undergraduate nursing students, as well as their interrelationships and key predictors. The findings showed that students reported a relatively high level of professional identity and academic satisfaction, while career commitment was moderate. Cluster analysis further identified distinct student profiles, with the most favorable group (only one-fifth) demonstrating high levels of all three constructs, while a substantial proportion of students exhibited either lower identity and satisfaction (one-third) or relatively high identity and satisfaction but weaker career commitment (two-fifths). Mediation analysis indicated that academic satisfaction partially mediated the relationship between professional identity and career commitment, with the direct effect becoming non-significant when the mediator was included. Multiple regression analysis further confirmed that both professional identity and academic satisfaction were significant positive predictors of career commitment, along with leadership role experience, while most sociodemographic variables were not significant contributors.

The present study found that nursing students exhibited high professional identity and academic satisfaction but only moderate career commitment. This suggests that while students strongly identify with the nursing profession and are generally satisfied with their academic experience, their intention to remain in the profession is comparatively weaker than their satisfaction. These results align with international evidence showing high professional identity and positive academic perceptions alongside moderate career commitment, especially in contexts offering alternative careers or migration opportunities (Wei et al., 2021; Wu et al., 2024, 2026; Ho, 2025). A recent systematic review identified professional identity as a key attribute among students with stronger nursing commitment, underscoring the importance of professional socialization throughout their education (Chen et al., 2025). A longitudinal study further showed that, while nursing students generally maintain positive professional perceptions over time, commitment levels vary across training stages (Ding et al., 2026). The moderate career commitment found in the present study aligns with the evidence that although graduating nursing students demonstrate positive but not uniformly high professional commitment, suggesting that factors beyond education influence retention (Tsai et al., 2026). The high academic satisfaction observed in this study is consistent with findings from a systematic review, where positive perceptions of the learning environment and faculty support correlate with satisfaction (Chen et al., 2025). Likewise, evidence supports that nursing education settings report moderate-to-high academic satisfaction, whereas some international studies document only moderate satisfaction among students experiencing academic stress and clinical learning difficulties (Abdi et al., 2025; Gazula et al., 2026). Findings from Iran also show favorable educational satisfaction linked to supportive learning environments and faculty engagement. Conversely, moderate satisfaction has been reported among students facing clinical challenges, academic stress and inadequate supervision (Abdi et al., 2025; Gazula et al., 2026). Collectively, these findings indicate that although nursing students develop a strong professional identity and academic satisfaction, sustaining long-term commitment to the profession remains challenging and requires ongoing educational and workforce support. This is consistent with the professional socialization theory which suggests that a well developed professional identity influences how students interpret their professional experiences. Nevertheless, a moderate career commitment suggests that positive socialization outcomes may not automatically translate into autonomous motivation.(Zarshenas et al., 2014; Sadeghi Avval Shahr et al., 2019).

The identification of three distinct student profiles highlights the heterogeneity of nursing students’ professional development and career outlooks. Similar person-centered analyses have shown that nursing students can be grouped into profiles characterized by varying levels of professional commitment, identity, and engagement, reflecting differences in how they experience and respond to nursing education (Tsai et al., 2026). The profile exhibiting high professional identity, high academic satisfaction, and strong career commitment is consistent with evidence suggesting that positive professional socialization and educational experiences contribute to a stronger commitment to nursing (Koch, 2014; Chen et al., 2025). Notably, the finding that nearly two-fifths of students reported relatively high professional identity and academic satisfaction but lower career commitment supports recent longitudinal evidence indicating that favorable perceptions of nursing do not necessarily translate into sustained career commitment, as factors such as sense of calling, career expectations, working conditions, and migration aspirations may also influence commitment trajectories (Chen et al., 2025; Ding et al., 2026). Furthermore, the presence of a substantial subgroup with low professional identity and academic satisfaction aligns with previous research identifying vulnerable student groups who may require additional academic, professional, and psychosocial support to strengthen their engagement with the nursing profession (Baldwin et al., 2014; Tsai et al., 2026).

Academic satisfaction showed a significant statistical indirect association in the relationship between professional identity and career commitment. This finding suggests that students with stronger professional identity also tended to report greater academic satisfaction, which was in turn associated with higher career commitment. However, because the study used a cross-sectional design, the temporal ordering of these variables cannot be confirmed. Therefore, this finding should be interpreted as statistical mediation rather than evidence that professional identity causes academic satisfaction or career commitment (Chen et al., 2025). This finding aligns with the growing evidence highlighting the importance of educational experiences in shaping nursing students’ professional development and career intentions. The significant indirect effect supports previous research indicating that students with stronger professional identities are more likely to experience higher academic satisfaction, which, in turn, enhances their career commitment (Koch, 2014; Ding et al., 2026). However, the relatively small indirect effect and low explained variance in career commitment (*R*^2^ = 0.088) suggest that career commitment is a multifactorial construct influenced by additional personal, educational, and contextual factors. This is consistent with prior systematic reviews that identified multiple determinants of professional commitment, including personal characteristics, social support, professional values, and workplace expectations. Therefore, while enhancing academic satisfaction may represent an important pathway to strengthen career commitment, broader educational and professional strategies are also required to support long-term commitment to the nursing profession (Chen et al., 2025).

Professional identity, academic satisfaction, and leadership involvement were significantly associated with career commitment among nursing students’ career commitment. The positive association of professional identity is consistent with evidence that students who develop a stronger sense of belonging to and identification with the nursing profession are more likely to report greater career commitment (Chen et al., 2025; Ding et al., 2026). Similarly, academic satisfaction was significantly associated with career commitment, supporting previous research indicating that positive educational experiences and satisfaction with the learning environment are linked to stronger professional commitment among nursing students (Ravi and Mohamed, 2022; Abdi et al., 2025; Gazula et al., 2026). Students who held leadership roles also reported higher career commitment, suggesting that greater professional engagement and involvement in leadership activities may be associated with a stronger commitment to the nursing profession (Tsai et al., 2026). In contrast, demographic and background characteristics, including age, gender, year of study, migration intention, and socioeconomic status, were not significantly associated with career commitment, suggesting that career commitment may be more closely related to professional and educational experiences than to personal characteristics. Nevertheless, the relatively low proportion of explained variance (Adjusted *R*^2^ = 0.091) indicates that additional factors not examined in the present study, such as sense of calling, resilience, clinical learning experiences, and workplace expectations, may also contribute to variations in career commitment among nursing students (Chen et al., 2025; Ding et al., 2026). Therefore, the significant predictors identified in this study should be interpreted as contributors to career commitment rather than comprehensive determinants.

### Strengths

The study employed well-validated and reliable instruments with strong psychometric properties to assess professional identity, academic satisfaction, and career commitment, ensuring the accuracy and consistency of the data collected. The relatively large sample size (*n* = 354) and inclusion of students from all 4 years of the undergraduate nursing program provided a comprehensive perspective on professional development across the academic levels. The use of advanced statistical techniques, including cluster analysis and mediation modeling, enabled a nuanced understanding of student profiles and the relationships among key variables.

### Limitations and recommendations

The limitations of this study include the use of a cross-sectional design, which restricts the ability to infer causal relationships among professional identity, academic satisfaction, and career commitment. Convenience sampling from only two nursing colleges, although geographically distinct, may limit the generalizability of the findings to all nursing students in India. Reliance on self-reported measures introduces potential response and social desirability biases, particularly given the moderate internal consistency of the Career Commitment Scale. Although the scale was retained because it is a short established measure and career commitment was central to the study objectives, the lower reliability may have attenuated observed associations. Future studies should consider using longer or contextually validated career commitment instruments among nursing students in India. Additionally, this study did not explore other potentially influential factors, such as clinical learning experiences, resilience, or workplace expectations, which may contribute to career commitment. The relatively low explained variance in the regression model indicates that important determinants of career commitment were not captured in this study. Future studies should include additional psychological, educational, and contextual variables, such as sense of calling, resilience, clinical learning environment, family expectations, workplace perceptions, and migration-related factors.

Recommendations include conducting longitudinal studies to better understand the causal pathways and changes in professional identity, academic satisfaction, and career commitment over time. Expanding the sample to include diverse nursing institutions across different regions and settings would enhance the representativeness. Incorporating qualitative methods could provide deeper insights into the contextual and personal factors that influence career commitment. Nursing education programs should consider targeted interventions to strengthen leadership opportunities and academic satisfaction, as these are significant predictors of career commitment. Furthermore, addressing broader motivational and systemic factors, such as career counseling, workplace conditions, and migration intentions, may be essential to foster sustained commitment to the nursing profession.

### Implications

The findings indicate that nursing students do not constitute a homogeneous group, necessitating tailored teaching and support strategies for their psychological well-being. Students with low professional identity and academic satisfaction may require additional academic and motivational support. Conversely, those with a strong professional identity but lower career commitment may benefit from increased engagement in the profession and more clearly defined career pathways. Academic satisfaction showed a significant statistical indirect association in the relationship between professional identity and career commitment, underscoring the necessity of a positive and supportive learning environment in nursing education. The regression analysis revealed that professional identity, academic satisfaction, and leadership involvement were associated with career commitment, whereas most background factors were not. This implies that enhancing educational experiences and providing leadership opportunities may be more effective than concentrating on student demographics to bolster commitment to the nursing profession.

## Conclusion

This study found that undergraduate nursing students reported high professional identity and academic satisfaction, but only moderate career commitment. Professional identity, academic satisfaction, and leadership involvement were significantly associated with career commitment. Academic satisfaction also showed a significant indirect association between professional identity and career commitment. However, the modest mediation effect and low explained variance suggest that career commitment is shaped by additional personal, educational, professional, and contextual factors not measured in this study. Future longitudinal and multicenter studies are needed to better understand changes in nursing students’ career commitment over time and to identify modifiable factors that can support long-term retention in the nursing profession.

## Data Availability

The original contributions presented in the study are included in the article/Supplementary material, further inquiries can be directed to the corresponding authors.
