# Supplementary material for: Professional identity, academic satisfaction, and career commitment among undergraduate nursing students in India: a cross-sectional study
Source: Front Psychol. 2026 Aug 19;17:1931177. doi: 10.3389/fpsyg.2026.1931177 (PMC13533743; doi:10.3389/fpsyg.2026.1931177)
Supplement: Supplementary file 1 [file Supplementary_file_1.docx]

# Data collection instrument

# Section 1: Participant Information

## **Section A: Participant Information**

**Instruction:** Please tick (✓) the appropriate option or fill in where required.

1. **Age (in years):**
2. **Gender:**
   ☐ Male
   ☐ Female
3. **Year of Study:**
   ☐ 1st Year
   ☐ 2nd Year
   ☐ 3rd Year
   ☐ 4th Year
4. **Area of Residence:**
   ☐ Rural
   ☐ Urban
5. **Reason for Choosing Nursing:** (You may select more than one)
   ☐ Personal interest
   ☐ Family influence
   ☐ Job security
   ☐ Unable to get another course
   ☐ Other (please specify): __________
6. **Do you intend to migrate to another country after completing your course?**
   ☐ Yes
   ☐ No
7. **Have you ever volunteered for social or community activities that are not mandatory as part of your studies?**
   ☐ Yes
   ☐ No
8. **Are you involved in any leadership roles in your college or university?**
   ☐ Yes
   ☐ No

# Section 2: Professional Identity Scale (PISNS)

| **slno** | **Item** | **Strongly Agree** | **Agree** | **Neutral** | **Disagree** | **Strongly disagree** |
| --- | --- | --- | --- | --- | --- | --- |
| 1. | I like to know more professional developing stories of some successful persons in nursing field. |  |  |  |  |  |
| 2. | I like to be a nurse. |  |  |  |  |  |
| 3. | I like my major and am positively preparing for my future work. |  |  |  |  |  |
| 4. | I am proud of working in the field of nursing. |  |  |  |  |  |
| 5. | Being a nurse makes me happy. |  |  |  |  |  |
| 6. | I am sure I will succeed in nursing field. |  |  |  |  |  |
| 7. | I have no plan to change my career direction. |  |  |  |  |  |
| 8. | Turnover will cause me emotional wounds. |  |  |  |  |  |
| 9. | I have already devoted too much to nursing career including economic aspect and effort, so I don't want to leave the field of nursing. |  |  |  |  |  |
| 10. | I like to communicate with predecessors of nursing field. |  |  |  |  |  |
| 11. | I try to know the condition of other career fields so that I can make my professional belief stronger. |  |  |  |  |  |
| 12. | I often explore my professional development by reflecting my interest, personality and values on myself. |  |  |  |  |  |
| 13. | Both one's own ideal and surrounding factors should be considered during the process of career choice. |  |  |  |  |  |
| 14. | I will choose the job I like no matter what other person says. |  |  |  |  |  |
| 15. | My knowledge about nursing career mainly came from parents, teacher and other relatives. *(reverse scored)* |  |  |  |  |  |
| 16. | Being a nurse can bring my creativity into play. |  |  |  |  |  |
| 17. | Being a nurse makes good use of my competence and advantages. |  |  |  |  |  |

**Section 3:Academic satisfaction scale**

| **slno** | **Item** | **Strongly Agree** | **Agree** | **Neutral** | **Disagree** | **Strongly disagree** |
| --- | --- | --- | --- | --- | --- | --- |
| 1. | I am satisfied with my current learning |  |  |  |  |  |
| 2. | The teaching methods used are effective |  |  |  |  |  |
| 3. | I feel engaged during learning sessions |  |  |  |  |  |
| 4. | The course meets my learning needs |  |  |  |  |  |
| 5. | I am happy with my overall learning experience |  |  |  |  |  |

**SECTION 4: Career Commitment scale**

| **slno** | **Item** | **Strongly Agree** | **Agree** | **Neutral** | **Disagree** | **Strongly disagree** |
| --- | --- | --- | --- | --- | --- | --- |
| 1. | I like this career too well to give it up |  |  |  |  |  |
| 2. | If I could choose a different profession that paid the same, I probably would take it. |  |  |  |  |  |
| 3. | If I could do it all over again, I would not choose to work in this profession. |  |  |  |  |  |
| 4. | If I had all the money I needed without working, I would probably still continue to work in this profession. |  |  |  |  |  |
